# Depoliticization of Violence: Critical Limits of Risk Environment Frameworks in Drug Use Research

**DOI:** 10.1177/00469580241308442

**Published:** 2024-12-20

**Authors:** Tyson Singh Kelsall, Jenn McDermid, Echo Vieira, AJ Withers, Vince Tao, Jackie Dives, Tara Myketiak, Nanky Rai, Jake Seaby Palmour, Mathew Fleury

**Affiliations:** 1 Faculty of Health Sciences, Simon Fraser University, Burnaby, BC, Canada; 2 Police Oversight with Evidence and Research (P.O.W.E.R.), Vancouver, BC, Canada; 3 Interdisciplinary Studies Program, University of British Columbia, Vancouver, BC, Canada; 4 Centre for Gender and Sexual Health Equity, Vancouver, BC, Canada; 5 Ruth Wynn Woodward Junior Chair, Gender, Sexuality and Women’s Studies, Simon Fraser University, Burnaby, BC; 6 Vancouver Area Network of Drug Users, Vancouver, BC, Canada; 7 Independent Researcher, Vancouver, BC, CA; 8 Department of Family and Community Medicine, University of Toronto, Toronto, ON, USA

**Keywords:** intersectional risk environment, health inequities, disablism, social epidemiology, liberatory harm reduction

## Abstract

The risk environment framework (REF) is a widely-accepted tool in policy research related to drug use. Its prevalence warrants a critical exploration of its strengths and weaknesses. This critical appraisal is a comprehensive analysis of the REF by definition and through relevant examples of its use within the context of public health evaluations, social science research, and epidemiological strategies in substance use-related policy. We examine the tensions inherent to a risk environment analysis, centering on its deficit-based lens of behavior, risk, and harm. This narrative review argues for shifts in frameworks within drug policy research away from individual- and community-level blame.

- This review examines use of the risk environment framework (REF) in drug policy research and governance. The REF has been a predominant tool in drug policy research for nearly two decades and its use warrants critical appraisal.

- This review unpacks the strengths and weaknesses of the REF, focusing particularly on how it can shield the systems and social relations that generate violence from reform and transformation.

- Lastly, we offer considerations of alternative research frameworks to employ in drug policy research that may be more effective at this juncture of a global crisis driven by a poisoned drug supply.

## Introduction

### Background

Drug-related policy analysis drawn through a “risk environment framework” (REF) offers insight into the multilayered factors that influence substance use and related behaviors.^1^ The REF has emerged as a dominant guiding framework in drug policy and health intervention research over the past 2 decades in many regions across the West.^2,3^ By examining the interplay of individual, social, and structural determinants, use of the model in policy research has had a role in shifting dominant public health discourse away from understanding drug use as a highly individualized problem to be managed, cured, and eliminated.^3^ As a lens, a risk environment can be useful in shedding light on some harms associated with drug use in a criminalized context; however, individuals and communities most negatively impacted by these forms of criminalization will generally be seen as contributors to the violence they are enduring within the definitional limits of a REF. Use of the REF has been prominent in challenging norms rooted in biomedical perceptions of drug use (eg, understanding any/all drug use as a medical disorder) and subsequent public policy literature. Likewise the REF has been instrumental in demonstrating the concrete health benefits of risk mitigating interventions in reducing the spread of disease and blood-borne infections (eg, HIV, HCV), as well as overdose—this includes the utilization of safe consumption sites, ensuring access to harm reduction equipment (eg, syringes, cookers, pipes, condoms, etc.) and increasing the availability and usage of low-barrier health and ancillary services in many settings across North America.^1-3^ However, even with these positive attributes, it remains essential to interrogate the limitations of the REF as a research framework, both because of its prevalent use, and due to the fact that wide-ranging harms linked to drug prohibition continue after 20 years of its dominant application. Our present critical, narrative review explores these limits and offers alternatives rooted in transformative-emancipatory approaches to drug policy research.

### Defining a Gap in Drug-Related Policy Research

Rhodes has defined the REF “as the space—whether social or physical—in which a variety of factors interact to increase the chances of drug-related harm,”^2^ focusing initially on the spread of HIV among drug users. Rhodes^1,2^ identifies 4 environments (social, political, economic, and physical) across micro and macro contexts to demonstrate the way in which the responsibility of “risk” extends beyond the individual to include a variety of environmental factors, pointing especially to the way in which micro and macro environments often interact to produce or reduce drug-related risks and outcomes. In this way, the REF highlights the “. . . dynamic and relational interaction between individuals and their environments,”^3^ and suggests that, while drug users have agency within their given environment, this agency is shaped by broader structural inequities (eg, poverty, criminalization, state surveillance, etc.).^1-3^ More recently, Collins et al,^3^ have expanded on Rhodes’ initial conceptualization of the REF to apply an intersectional lens to this framework. Coined by legal scholar Kimberlé Crenshaw, use of the intersectional paradigm recognizes the multi-level interacting social locations, forces, and power structures that inform and influence drug user’s lives. Intersectionality promotes an understanding of human beings as shaped by the interaction of different positionalities (eg, race/ethnicity, gender, class, sexuality, geography, age, disability/ ability, religion, etc.) and power and structural forces, through the examination of multiple axes of privilege and oppression.^4^ Broadly, Collins et al’s^3^ widely-cited work incorporates intersectionality with the intention to deepen understanding of how social locations (eg, race, gender, class, etc.) converge within the risk environment to shape drug users’ experiences across different settings. Given the considerable influence of Rhodes^1,2^ and Collins et al’s^3^ work within research and policy settings related to drugs, we have chosen to focus this paper on their seminal works.

Risk mitigation-oriented research has challenged the way in which drug use(rs) are conceptualized and understood within public health domains, as well as drawn important connections between risk mitigation interventions and subsequent health benefits. However, REF-based research and policy does not interrogate the social relations and unjust systems of power that generate and normalize the mass violence endured by drug users. By not inherently challenging systems of power and violence, the burden of responsibility of drug prohibition, systemic criminalization, and their connection to the logics of settler colonial control and racial capitalism have been diminished in dominant policy discourse related to drug use. The most widely harmful and violent components of prohibition include the international drug toxicity crisis^5^ and racist, mass incarceration across North America and much of the West.^6^ For these reasons, our review and appraisal intends to outline gaps in the REF as a guiding framework in policy research related to drug use(rs), and offer alternatives by shifting focus instead toward transformative-emancipatory paradigms, including abolitionist and anti-colonial approaches.

The risk environment analysis shapes the gaze of research to focus on how collective behaviors of people who use drugs can increase public health harms in relation to how people interact with their environmental at-large. While we have outlined how risk environment analyses can support in demonstrating the health benefits associated with risk mitigating strategies, by definition research that employs a REF tends to provide support for interventions that may mitigate specific, present-day behaviors, rather than examine the intersectional social relations that *generate* and *normalize* violence and increase risk. This is particularly important as the mechanisms of social relations (eg, the bureaucratization of drug user violence, social exclusion, racist and imperialist approaches to drug policy, the scapegoating of drug use(rs) for a wide array of social disorder, etc.) can reinforce and normalize the violence and harm perpetuated against drug users on a structural and systemic level, creating the conditions in which incrementally worse forms of violence (eg, the mass death caused by prohibition and the subsequent worsening of the toxic drug supply) persists as routinized and justified. While we recognize that pointing to risk behaviors helps to reduce drug-related harms on a micro and, in some cases, a macro level,^2,3^ it does not identify and address the social and power relations that allow for the violence endured by drug users to be broadly seen as status quo, and subsequently maintained.

This narrative review and critical reflection examines the limitations of the REF in policy research and methodologies related to drug use(rs), drawing primarily on three core texts, two by Rhodes^1,2^ and one by Collins et al^3^ We also draw from relevant examples of the REF being applied in research, and other related risk-focused analysis. We first unpack the limits of the REF and outline the ways in which the REF’s narrow focus and widespread use has shaped understandings of drug user self-determination and stunted the advancement of drug policy research. We then offer a discussion around alternative frameworks, with a primary focus on abolitionist and anti-carceral theory. We suggest that such a transformative methodological and cultural shift in drug research and policy is necessary to better identify the racist, imperialist, socioeconomic and carceral logics that continue to underpin drug policy reforms in North America, and will ultimately offer a vision for the future that moves beyond punitive approaches to drugs and drug use.

### Risk Environment Framework in the Context of Organized Abandonment

We write this critical, narrative review in the context of heightened organized abandonment^7^ of drug users, and the poverty governance^8^ of people who use drugs most visibly across North America and other international jurisdictions, including through involuntarily displacement and/or embedding law enforcement into health and social services.^9,10^ These forms of social regulation contribute to conditions where the risk of using an illicit substance has become a matter of life and death in many settings. Notably, this acute form of violence is consistent with the intent of the socioeconomic logics and relations that benefit from it. As risk mitigation-oriented research and policy rarely challenge systems of power, the burden of responsibility on drug prohibition, systemic criminalization, and their connection to the logics of settler colonial control and racial capitalism have been diminished in dominant drug policy discourse. As risk-focused research does not generally direct a primary focus toward the dismantling of unjust social relations, institutions and/or the re-organization of power, risk mitigation (ie, compromise) strategies have an inherent limit to envisioning futures with alternative or renewed relationships to drug use, procurement, ritual and production. This is particularly pertinent when primary research itself is not connected to a broader social movement. There exists a litany of literature on what constitutes a “drug” as shifting (or becoming medicine) across time, cultures and political systems.^11-13^ Use of the REF opens up conceptual challenges to notions of substance use harm as an individualized problem, but continues to exclusively explore negative outcomes related to drug use within research methodologies and analysis by its own definition. As Larney et al^14^ suggest, drug policy research has “been narrowly focused on harms and ‘risk behaviors’, with insufficient attention to non-harmful–even beneficial–outcomes of drug use.” We argue that the omission of a substantial critique of overarching power relations hinders transformative visions of drug policy.

We draw on insights from abolitionist and anti-colonial theory to fill the gaps left by REF parameters and as alternative frameworks for policy analysis that governs drug use and possession. In incorporating abolitionist and anti-colonial theory, we are arguing for the need to move away from an ecological paradigm underpinning Rhodes and Collins’ work, toward a more transformative-emancipatory research paradigm that synthesizes different forms of knowledge and creates the possibility for meaningful social-structural and systemic change to reduce violence and premature death. Employing these approaches allow us to envision the eliminating drug prohibition and its interconnected forms of surveillance and punishment. Abolitionist theory and practice directly challenges violent and carceral systems, while seeking to dismantle them.^15-17^ Drug law is typically entrenched in racist carcerality, including border violence,^18^ whereas abolition envisions an “antiracist tomorrow.”^13,19^ Drug prohibition also serves as a caretaker to forms of settler colonial power^20,21^ racial capitalism (ie, an understanding of capitalism that does not separate the histories of racism and economic exploitation in the West),^22,23^ and disablism.^24,25^ This article demonstrates how the parameters of a risk environment not only fail to holistically examine injustice, but that what the framework constructs may perpetuate it; and we contend that drug policy futures should be grounded in abolitionist and anti-colonial principles to effectively address punitive, colonial, racist, and disablist drug war harms.

## Limits to the Risk Environment Framework

### Systems of Violence: Settler Colonialism, Racial Capitalism & Criminalization

Collins et al^3^ define risk environments as “the social or physical space in which risk and harm are produced or mitigated by the interplay of factors exogenous to the individual.” These parameters can be useful in research; however, the sharpened focus on “interplay” between people and their environment restricts examination on the *production of violence*, particularly regarding the roles of settler colonialism and racial capitalism. This is due in part to the focus on behaviors embedded in the definition of the REF, rather than a focus on the producers of violence (ie, policy, institutions, relations of power, beneficiaries of a status quo, etc.) and how they can be dismantled or effectively reformed to reduce violence. Within a REF definition/perspective, researchers must continue to consider behavior as a contributor to the crisis of prohibition, which obscures *who* and *what* is responsible for systemic-level “risk,” leading to results that suggest minor changes, which is not necessarily proportional to the harm prohibitionist policies are causing toward the general public. This absence neglects to interrogate *who* a state functions to protect against premature death and other forms of harm, and who is treated as disposable.^26,27^ Drug war logic and prohibition are successful on the basis of their intent, which is rooted in “state power, capitalist accumulation and social inequity.”^26,28^ Through its focus on interactions and reactions between people and systems, risk mitigation research tends to function under the assumption that a state will seek to reduce risk/harm across populations if provided with rational methods and corroborating evidence for doing so. However, in many regions, inertia and/or explicit, ongoing settler colonialism and racial capitalism signify that violence is embedded in its function, including in relation to drug use.^12,20,28^

When race is measured as a primary risk factor while employing a REF, the logics of racial capitalism are often concealed. For example, Cooper et al^29^ position “racialized” groups/communities as “experience[ing] different living environments” (p. 44), which is a passive framing. This perspective implies that a “racialized risk environment framework” can be employed to position racialized communities as responsible for the adverse social outcomes that are, in reality, produced by settler colonialism and racial capitalism, which engender social malaise and inequity. Collins et al^3^ adopt a more intersectional lens when considering race and drug use. However, they conclude that “how race intersects. . .across environmental dimensions is imperative to addressing and mitigating health outcomes for racialized” people who use drugs (p. 5), rather than focusing on undoing racial capitalistic relations and accompanying forms of violence. Collins et al^3^ cite Crenshaw’s^4^ definition of intersectionality; however, Crenshaw’s work is fundamentally an interrogation of resistance strategies, including within legal theory and political struggle, which serves as an analytical tool for creating more effective coalitions aimed toward undoing violent social institutions (p. 1299). Crenshaw^4,30^ was not gesturing in mere acknowledgment

of the way harm could be categorized, especially regarding anti-Black gendered violence, but rather building “hands-on tools that advocates and communities can use” to better intervene.^31^ The crucial aspects of untangling and resisting these forms of power are largely absent within risk environment parameters. This absence aligns with Bilge’s^30^ critique that intersectionality has been “depoliticized” and “commodified and colonized for neoliberal regimes,” including within academic institutions (p. 407). Collectively, this depoliticization permits and/or compels risk environment-based analysis to describe state-produced harm caused as “unintended” or as “unintended consequences” (eg, Refs.^1,32-37^) or perceive policy-induced harm as state failures, rather than recognizing the integral role of settler colonialism, racism, disablism, cisheterosexism, and anti-drug user biases embedded within states—that is, *the system isn’t broken, it was built this way*.^38^

Use of biomedical risk-focused research frameworks likewise tend to articulate the drug toxicity crisis as apolitical and naturally occurring by describing overdose death as passive phenomena; for instance, referring to the crisis as “driven primarily by unintentional opioid toxicity deaths resulting from the use of a highly adulterated unregulated drug market”^39^ (p. 2). This type of framing remains focused on use as individual behavior, rather than implicating prohibitionary policies as drivers of these conditions.^40^ Related research likewise inaccurately and vaguely differentiates carceral approaches from healthcare practices.^33,41^ This separation obscures the violence drug users face within healthcare settings, which are often formally linked to law enforcement bodies and experienced as surveillance-oriented, racist, traumatic and/or unhelpful space.^42-47^ Wohlbold and Moore^48^ posit that “maintaining this false binary between health and criminality is the most telling feature of benevolent whiteness” in drug policy (p. 25). Some risk environment analysis does characterize healthcare as a possible sites of harm for drug users, particularly for individuals who experience multiple forms of oppression.^3,49^ This tension exists partly due to who is included in developing causal relationships within the results in drug policy research. For example, researchers often work within systems that are complicit in violence related to the criminalization of drugs. This can include academic and health research institutions typically steeped in settler colonialism,^50^ whiteness,^51^ and adjacent norms of sobriety.^52^ It is therefore unsurprising when risk environments neglect to document or contest these relations, as those with proximity to current iterations of power are deemed to be the suitable negotiators of drug policy. This is despite drug using, racialized, and/or Indigenous individuals and communities facing the brunt of harm produced by the drug war and associated logics of imperialism.^13,27^ Neglecting to criticize these major sources of power depoliticizes evidence, which in turn sees political actors integrate evidence on an ad-hoc basis, without organized resistance or accountability. This is furthered by the fact that such institutions systematically benefit (through funding, academic prestige, funding, etc.) from the impacts of prohibition-based policies, an incentive to research and the recommendation of approaches that mitigate harms associated with the status quo, rather than work toward modes of radical transformation. Substantially undoing systems of violence would render mitigation-focused research less salient. and therefore less attractive to funders, which is a systemic conflict of interest.^38^ Moreover, as community-based capacity building is not an inherent methodological component to the REF, researched communities might be left out entirely of research benefits, including attached funding, from the data extracted when evidence is ignored or misused.^38,53,54^

### Autonomy of the User: Intersectional Versus Intercategorical

When interactions between people using drugs and their environment is positioned as a precursor for “disease distribution”^3^ (p. 1) or as a risk to contribute “immensely to the global burden of disease,”^55^ (p. 1), use of a REF can perpetuate fear and the scapegoating of drug users and/or people who live with duel realities of blood-borne infections (which is frequently measured as a “risk”) and structural violence, and tend to suggest behavioral interventions to reduce “risk,” rather than directing responsibility toward the prohibitionist policies that generate substance use-related harm, discrimination in healthcare settings and social isolation. Previous risk environment analysis has incorrectly assigned the cause of mass deaths from the drug toxicity crisis to the overprescribing of opioids in some jurisdictions,^56^ and on the relative potency of fentanyl and other opioids (ie, Refs.^57-59^), not the policies and decisions that uphold prohibition of drugs that generates an unpredictable, increasingly potent and complex drug supply, containing more elements than solely substances that are primarily opioids.^40,60-64^ This is reflected in media and public perceptions of all forms of fentanyl as reduced to a monster drug to be feared, despite its multifaceted use.^65,66^ Drugs are neutral and non-sentient, and it is rather governance, policy and conventions that shape potency, availability and harms.^11,40^

**Figure 1.**
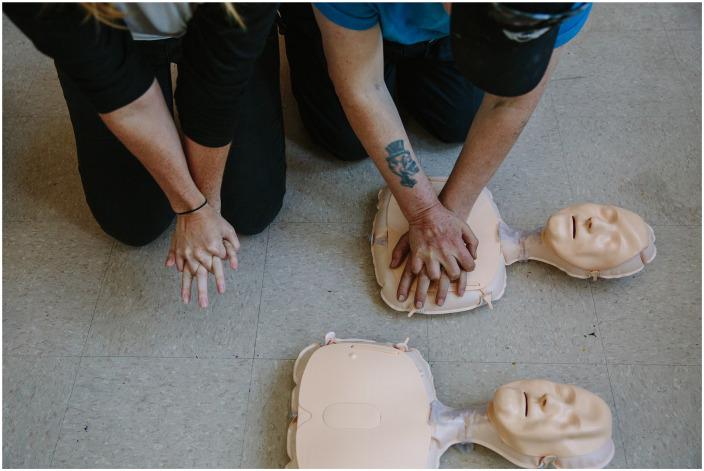
Vancouver Area Network of Drug Users opioid overdose response training, an example of “acts of community care.” Photo by Jackie Dives.

The parameters of a REF analysis discursively reduce the agency/control drug users have over their healthcare, by coding and pathologizing people who use drugs as diseased, needing help or to change themselves, and proposing services to mitigate risk and reform behaviors, rather than building services led by and alongside drug users. While environmental and socio-structural contexts may interact to shape behaviors and experiences of drug users, people who use drugs, including those that experience marginalization, are not a homogenous group.^67,68^ Quantitative drug use/policy research often draws from determinations of statistically significant or seemingly important relationships, and qualitative approaches utilize theory to frame causal processes. Causal exploration is therefore an essential step in knowledge translation. When analysis, primarily quantitative, is understood through discourses of risk, causality skews intra- and inter-categorical rather than intersectional; in other words, it measures specific outcomes of social groups burdened with multiple forms of marginalization^69^ (p. 495). However, categorical analysis does not “delineate the distinct mechanisms” that drive health and social disparities^69^ (p. 495). This can deny multiplicity by “concretizing” forms of identity that are in part defined by their relation to norms and context, including gender^70^ (p. 193), disability,^71^ and drug user status.^72^ Lapalme et al^69^ pinpoint the crux of this issue: “Social inequalities in health research have mostly continued to focus on differences between social groups without empirically accounting for the social processes that have shaped these differences” (p. 495). DiNova^73^ and Smith^74^ have positioned this form of essentialism as part of constructing “inferior” populations in Western research. Metrics of health disparities and inequity among categories of racialized peoples are as much a measurement of harm as they are a part of the construction of what *racialization* is.^40^ This is reflected in the widespread conflation between daily (or the pathologized term, “disordered”) substance use and drug toxicity deaths, even though the crisis impacts people with diverse relationships to drug use, including those who use illicit drugs occasionally,^12,67^ or those who do not otherwise meet criteria for diagnosis,^67,75^ These conflations are also replicated in labor roles, pay and power in drug policy and overdose response work, with drug users often viewed as inferior knowledge holders.^52,76,77^ Acts of community care, such as in Figure 1 below,^78,79^ joy and euphoria,^12,80-84^ modes of resistance,^85^ and/or grassroots efforts to uphold drug users’ safety^79^ are also largely unaccounted for when employing a REF, effectively erasing drug users and drug user movements in analysis and strategy. The tendency of the REF to essentialize drug users and/or construct “the drug user” as a very specific groups of individuals despite wide use (Das and Horton^5^; Death Review Panel^67^) works to deny the positive impacts that drugs can have in people’s lives including the pleasurable, relaxing, and social aspects. While it is widely understood that drug use has benefits as well as risks, REF-based research, by definition, prioritizes measuring adverse drug-related outcomes.^2,12,86^ Gordon and Webb^87^ connect increased focus on risk and “strategies for addressing the *problem”* with aims to “contain” risk rather than impact change (p. 280).

Likewise, Gentry^88^ outlines how the construction of “at-risk environments” shapes understandings of systemic violence as confined to a specific geographical area, and that while “the theoretical model [describes] the vicious cycle facing those adversely impacted,” it “lacks an interpretative paradigm that demonstrates how race, class, and gender” can be targets of violence (p. 244)—as is likewise true regarding sexuality and disability. According to Rhodes,^2^ the REF aims to solely to reduce risk “at the *community level*” (p. 86). Gentry^88^ argues that analysis should better center Black feminist thought “to question the legitimacy of those social institutions that exploit black women or any other oppressed groups in our society” (p. 245). Furthermore, Gentry^88^ draws parallels between the label of “high-risk lifestyle” (p. 246) with being “accused of causing social problems” (p. 246). This depoliticized form of risk reduction categorizes these types of violence as spatially-, community- and population-confined deviance, freeing the state from the “obligation to address the underlying social and economic conditions” that generate violence and drug market harms^88^(p. 246). While epidemiology has a role in targeted outbreak analysis, drug poisonings are a systemic *social crisis* produced by governance, not a harm confined to a specific time, place and/or person. Risk environment logics are applied to uphold “facilities such as homeless shelters, drug treatment facilities, and halfway houses. . .constructed for high-risk persons to legitimize their removal from spaces more desired by the middle class”^88^ (p. 251). These forms of displacement and separation from wealthier areas, while poor residents are portrayed as the percolator of social issues, are replicated in many geographical areas, such as the Downtown Eastside in Vancouver and Los Angeles’ Skid Row.^89-92^ The impact of painting issues as more spatially confined than they are can flow in multiple ways: both by expanding carceral-oriented policies within a “problematic” neighborhood to “contain” risk, while communities outside these spaces perceive themselves as immune to contributing to or enduring the same violence. This confines autonomy by limiting mobility (class, spatial, and social) among members socially coded as being part of these communities. This is further reflected in the discursive separation between social or public policy and “drug policy,” even though the latter tends to impact people beyond only those coded as drug users (ie, through expanded police discretion, governance of public spaces, access to medication).

### Focus on Harm Obscures Structural Violence-Free Visions

The focus of public health analysis produced and filtered through the REF is centered on a recognition of harms, and the implementation of strategies to mitigate or contain them. It is by this logic that in Western, utilitarian-based public health models, the REF is used to shape harm reduction interventions as confined to a single setting, rather than as a movement for social change. While hyperlocal and contextually attuned, these public health analyses predominantly revolve around behavior, rather than state obligations, violence and/or provision of positive rights. Consequently, recommended interventions are often bereft of societal factors that contribute to the construction of criminality, the tangible repercussions of policing and criminalization (brutality, harassment and mass incarceration), and the intricate sociopolitical dynamics that delineate permissible ways of being.^38^ These oversights manifest as neglect and underscore the impacts of community exclusion from knowledge production and benefits sharing in research. Limiting the parameters of discourse about what harm reduction is severs it from its radical history.

**Figure 2.**
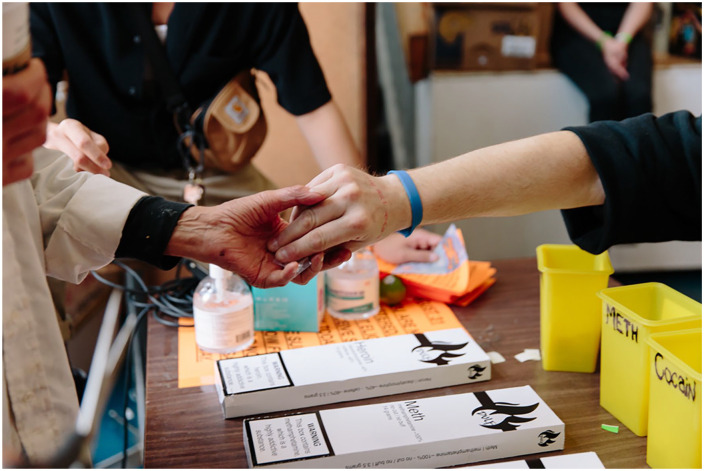
Community distribution of tested and labeled drugs. Not one member of this compassion club died while it operated during a formal, overdose public health emergency (Kalicum et al^95^). It was raided and shutdown in October 2023. Photo by Jackie Dives.

These discursive limitations, in turn, constrain analytical possibilities for alternative futures. Boilevin et al^93^ have outlined ethics of research on communities to include reciprocity and “actual power” for impacted populations within research projects (p. 18). Rhodes and Lancaster^94^ have highlighted the potential of a “*different mode* of drug policy research. . .inviting of multiple alternative future possibilities.” In order to address violence directed toward drug users, it is required that we engage in strategies that dismantle the institutions and social relations that underpin and sustain drug user harm, while at the same time building up networks and relations of support that enable communities to flourish. Figure 2 presents one photographic example of a speculative alternative led by the Drug User Liberation Front, who operated a community-run compassion club model, testing and labeling illicit substances prior to distribution in Vancouver, BC, until it was raided in October 2023.^95,96^ The action taken by the Drug User Liberation Front represents a real transformative, liberatory approach to drug policy by undermining the state violence reproduced by prohibition and providing access to safe, regulated alternatives. Mitigation strategies do not capture the speculative potential of undoing violence through policy frameworks, such as drug legalization and regulation with social equity initiatives attached; the defunding and abolition of police and penal institutions; or the state provision of positive rights, such as providing dignified housing for all. Additionally, Schiele^97^ has contrasted dominant Eurocentric approaches to substance use issues as linked to individual-level efficiency and productivity, with an Afrocentric paradigm that focuses on common goals (rather than individual differences), creating space for demands for change. Some risk environment research calls for the opposite of by recommending the entrenchment of institutions that uphold the status quo, including the integration of police and coerced treatment with harm reduction services,^98^ and expanded resources/tools for police officers.^33^ Other instances of REF-based research will recognize major sources of violence, such as in borders and deportation (ie, Goldenberg et al^99^), but recommend only that those “trajectories should be further explored” (p. 1190), and focus on the narrow recommendations of local health programing.

### Public Health Advances & Sanitation of Harm Reduction

The REF has been utilized in practical advocacy in support of life-saving and life-affirming health interventions (ie, Refs.^100-102^), alongside drug user-led groups (ie, Refs.^79,103^). Harm reduction interventions produced through a risk environment framework, such as safe consumption sites in particular, can act as “a space of autonomy within” a context where drug use is highly criminalized.^104^ This vein of research has importantly established broad scholarly agreement regarding the harms caused by a criminalized and unregulated drug supply, through explicitly discriminatory policies (ie, Refs.^105,106^) and those embedded in our social fabric (ie, Refs.^103,107^) However, research that continues to apply a REF analysis risks re-producing concepts and structures that are, in some cases, antiquated and unnecessary, including in the flow of material resources connected to research. Notably, a risk environment lends itself to situating harm reduction as a public health intervention and as mitigation-based service delivery, rather than a social movement strategy that envisions a decreased need for harm reduction itself (ie, regulation and de-medicalized access to drugs), because it focuses on interventions rather than systemic change. State refusal to meet crises driven by carceral drug policy with appropriate urgency, including intervening to reduce the unpredictability of the illicit drug supply, can be understood as a “sanctioned massacre”^85^ (p. 78). In Canada for example, there has been a dire lack of immediacy in addressing the over 45 000 prohibition-related drug toxicity deaths, despite evidence that legal regulation would significantly reduce the harms perpetuated by the unregulated drug supply. In many other contexts, a death-rate this high would result in swift and immediate systems-level change that moves beyond piecemeal public health interventions.

Despite its origins as a grassroots movement that opposed state and legal authority, harm reduction has become sanitized and depoliticized through public health institutionalization and the nonprofit industrial complex (ie, nonprofits being accountable to revenue and wealthy funders, regardless of community need).^24,38,107^ Moreover, the nonprofit industrial complex can siphon resources away from community aid, and entrench status quo arrangements of power.^38^ Harm reduction originated as a liberation practice, where, for example, at the height of 1980s AIDS activism, community members, and politicized frontline workers organized for structural social change and risked arrest to distribute sterile syringes and other harm reduction supplies as a means of reducing the harms associated with drug use in a criminalized context.^85,108-110^ Signaling a set of values in individual and societal responses to drug use and drug markets that views drug use as an inevitable fact rather than moral issue, harm reduction emphasizes humanism, self-determination, and the rejection of both abstinence as a precondition to service access and criminal law as a tool for the governance of drug use.^46^ Morgan^24^ suggests that liberatory harm reduction as practice should be steeped in joy. They contend that liberatory harm reductionists:

. . .support each other and our communities without judgment, stigma, or coercion, and we do not force others to change. We envision a world without racism, capitalism, patriarchy, misogyny, ableism, transphobia, policing, surveillance, and other systems of violence.

In its institutionalized form harm reduction “is used by public health institutions as a descriptor to characterize programs and services that advance population health goals of reducing disease burden among specific priority populations”^46^ (p. 5). In other words, institutionalized harm reduction focuses on service delivery and its quantitative metrics. This dilutes the political origins of harm reduction, while also refusing to confront the things that produce the most significant harm for drug users: prohibitionist drug laws and broad structural inequities.^109,111^ Roe^91^ and McLean^85^ trace the institutional co-opting of harm reduction as coinciding with rises in social disorder policing, and reduction in state allocation toward health and legal services. Institutional harm reduction/reductionists inherently practice harm reduction as a top-down policy rather than recognizing it as a bottom-up movement, and the social issues they intend to address are also created and perpetuated by the social relations in which they work.^85,91^ Roe^91^ outlines how institutional harm reduction reflects use of a REF analysis as a novel form of control designed to “minimize risk from, and maximize control over, marginalized populations such as drug users” (p. 245). The logic of only measuring outcomes of harm and risk can be and is utilized to justify the expansion or adaptation of carceral, settler colonial aspects of the state used to contain and control risk (and those labeled risky).^87^ As this direction is congruent with the status quo, political inertia and/or intent propels risk environments to be applied toward carceral growth more often than used to dissemble the machinery that generates risk and harm.

## Abolitionist and Anti-Colonial Alternatives

The contemporary make-up of carceral power that impacts drug users is rooted in interlocking structures of settler colonialism,^112^ racial capitalism^13^ and disablism^24^; social relations that frequently intersect across the colonized world.^38,113-115^ International cultural and contextual experiences of ongoing colonialism are far from homogenous, and essentialist theorizing can be problematic^74^; however, Chartrand and Rougier^112^ delineate a thread across these distinctions, in that “the formation of the world today has deep and widespread roots in a European colonial logic. . .European colonization pervaded more than three quarters of the world’s land mass by the mid-20th century” (p. 22). These theoretical overviews deserve extrapolation depending on research context and setting, including time and location. In general terms, it is understood that Western carcerality is deeply rooted in “colonial conquest”^112^ (p. 28). Chartrand and Rougier^112^ theorize that for abolition “to be as equally creative and fluid [as carcerality], it must account for these deep colonial roots and denaturalizing relations of the carceral apparatus.” Police and the carceral partnerships or provision in state “services” are the frontline of colonial power, reinforcing the interconnectedness of these violent systems worldwide.^116-119^

### Abolition/Creation in Drug Policy Research

Contemporary abolitionist theory is rooted in active anti-carceral social movements. These movements strive to work toward the abolition of all carcerality within the state and its overarching social relations; this includes the abolition of police and prisons. Abolitionist theory and practice, driven by Black feminist leaders and communities, connects slavery with the violence upheld by pervasive carceral systems central to many nation-states in slavery’s afterlife.^17,120,121^ Abolitionist frameworks directly confront present-day state violence while aspiring to foster a more just future.^15,16^ Halle-Erby and Keenan^16^ write that “abolition-compatible methodology requires. . .training our gaze upon social structures that are commonly taken for granted” (pp. 133-134). The abolition of state violence would be liberation from it. Abolitionism scrutinizes actions and resource distribution that reinforce carceral infrastructure, in recognition that racial capitalism and settler colonialism are central to the carceral project,^90,120^ and aims for a world devoid of these relations of violence and their consequent carceral institutions.^17,38,121,122^ Abolitionism is inherently action-oriented, and rejects passive theorizing by supporting “insurgent practices already underway”^16^ (p. 134), which likewise bleeds into research methodologies. The nature of appropriate contributions is contextually shaped and oriented. We contend that abolitionist theory can be applied to contesting all drug policies intertwined with carcerality, often exemplified by the settler colonial and racist drug war, prohibition, collaborations between medical models and carceral systems, border violence, and incarceration rates. Abolitionist social science research is emergent within academic literature, but has long existed outside of it. The academy itself “has its own history of contributing to carcerality” and questions remain on how that could limit abolitionist methods existing within it^16^ (p. 135). By definition, abolitionist methods necessitate participatory methods and capacity-building, driven by a more utopian objective of rendering top-down research and interventions related to social issues obsolete.

Abolitionist approaches to research cannot exist without an integration of a disability justice lens. Carceral logic is in part applied through the containment of non-normative behavior, and disability is broadly understood and constructed through its relationship to normativity. While risk environments advocate context-specific accommodations, abolition demands accessibility for all by confronting what would otherwise be state-produced harm. In Collins et al,^3^ the authors suggest future examination on drug users practices with “varying abilities” (p. 5) through an intersectional REF. While examining access needs of individual drug users and the health needs of disabled drug users is important, Collins et al^3^ individualize and essentialize disability through this framing. As articulated by Withers,^71^ “disability is a social construction used as an oppressive tool to penalize and stigmatize those of us who deviate from the (arbitrary) norm” (p. 98). Further, using the language of “ability” reifies disability as a biological reality rather than a social construction.^115^ Like the REF, deficit-based assessments of “ability” focus on populations; whereas, a disability justice analysis and abolitionism focus on implicit and explicit socially exclusionary policies and practices. Moreover, disablism drives carceral expansion, not only in association to prisons, but also psychiatric hospitals and other forced/coerced residential institutionalization.^45,123^

Furthermore, research incorporating the REF often includes recommendations made to the same institutions, including governments, that produce drug use-related harms. This is antithetical to an abolitionist theoretical framework, which contends that the same social relations responsible for the oppression of drug users cannot be the same ones that will be responsible for drug users’ justice/liberation.^124,125^ Researchers applying a REF have often suggested that, in order to protect drug users from the harms associated with the illicit drug toxicity crisis, it is critical that health systems are centered in the expansion of services, sites, and programs that promote drug users’ safety (ie, the adoption of harm reduction services primarily as medicalized settings, physicians gatekeeping of regulated opioids, etc.). This is despite a well-documented and ongoing practice of medical practitioners and bodies working to undermine drug users’ autonomy and well-being (ie, labeling drug users as “drug seeking,” surveilling drug users, stopping prescriptions with little or no warning, refusing to listen to drug users as experts in their own care, etc.).^22,48,82^ While drug users should receive dignified and comprehensive care *within* health settings, such solutions will not change drug users’ material conditions or remove the fatal risks driven by the impacts of prohibition. Applying an abolitionist theoretical framework requires demands for the radical transformation of violent policies and practices directed toward drug users, particularly the elimination of prohibition.

### Anti-Colonial Strategy in Drug Policy Research

Anti-colonial thought contains aspects of connecting to histories, knowledges undergoing colonial silencing,^126^ and creates opportunities for alternative futures. Anti-colonial research approaches are concerned with present-day solidarity among colonized peoples while envisioning worlds and future emancipated from colonial power. Anti-colonial strategies firmly reject the normalization of settler colonialism, while striving to deconstruct it. At its core, an anti-colonial framework represents a direct confrontation against the hegemonic relations of colonialism. Fundamental to anti-colonialism is the reclamation of spaces colonized by settler colonial power.^127^ Daniels et al^27^ have situated uncoupling drug control and criminalization, as well as divesting from related law enforcement costs into community care as part of decolonizing drug-related policies. Research approaches aligned with anti-colonial principles should cultivate processes that respect and privilege Indigenous worldviews and epistemologies.^74,112^ Moreover, anti-colonial methodologies accentuate the agency and self-determination of colonized peoples. By building solidarity and challenging settler colonial power structures and legacies, anti-colonial research seeks societal transformation and aspires to construct a more equitable, inclusive, and decolonized future.

### Relational Models Have Always Existed

Although not acknowledged by Rhodes^1,2^ or Collins et al,^3^ the REF integrates many existing aspects of anti-colonial, Indigenous and decolonial theories, such as: viewing layered social, economic, and physical (as well as spiritual) relations as interacting^50,74^; understanding individuals within/embodying broader relations^50,74^; stratification of inequities across different subpopulations^128,129^; and interactions^130^ between systems and individuals as a fluid process.^50^ Recognizing the limited discussions on future-oriented drug policy research, Rhodes and Lancaster^94^ more recently suggest inclusion of “relational elements in ecologies of drug futures” (p. 1).

While Rhodes and Lancaster^94^ appear to be opening the analytical gates by contending that “speculative drugs policy research is warranted” (p. 2), these elements have long existed within anti-colonial theory from the racialized and/or Indigenous scholars that are unacknowledged in the core REF texts. Anti-colonial, decolonial and abolitionist methodologies have been partly defined by imagining alternative futures.^19,74,128^ Anti-colonialism and abolitionism both imagine worlds liberated of oppressive systems, including settler colonialism, racial capitalism, and state produced violence. Furthermore, anti-colonial theory has “always. . .engaged in social and political relations”^128^ (p. 300). Rhodes et al^130^ theorize that “risk environment can give insufficient attention to Nature[sic]” (p. 1) and posit a need to formulate another term, “ecological harm reduction” to address this. This replicates another semantic practice of harm reduction, while many existing anti-colonial and Indigenous methodologies position land and the natural world as interdependent with human geographies, or do not make a distinction between the two.^131-134^ Holistic understandings of relationality, sometimes beyond what the academy can or has articulated,^119,135^ are integral to what Indigenous, and anti-colonial methodologies have long contested in Western theories of knowledge.

## Conclusion

Violence caused by prohibition deserves urgent attention. Prohibitionist drug policies rooted in criminalization are violent and perpetuate significant harm. Central to this harm are the global drug toxicity crisis and pervasive drug-market violence; these realities result in mass deaths and lead to various adverse consequences, such as health complications, incarceration/criminalization, social marginalization, and interpersonal and institutional violence. The drug toxicity crisis, caused by these prohibitionist policies, and marked by the rise of more potent and unpredictable drug supply, has claimed countless lives and left communities devastated. Our critical reflection and analysis underscores an urgent need for immediate support and scaling up of emergent, liberatory harm reduction strategies and drug policy reform that centers on the legalization and regulation of all drugs people use, while challenging and undoing settler colonial and racial capitalist relations.
